# Knitting History Through Reconstruction: The Making and Meaning of Early Modern Stockings

**DOI:** 10.1080/00404969.2024.2393229

**Published:** 2025-01-16

**Authors:** Paula Hohti

## Abstract

Knitted stockings were one of the most important early modern textile innovations. Especially fine stockings made of silk were a popular fashion product and one of the key novelties among European elites. The popularity of knitted stockings is seen in the fact that by the end of the fifteenth century, there were thousands of professional knitters in Europe. Despite the prominence of knitted stockings in this period, there is little surviving documentation about actual knitted stockings and their early histories of making. What exactly made knitted stockings so luxurious and appealing for early modern men and women? This article combines visual and textual evidence, close reading of surviving objects, and scientific analysis with historical reconstruction, in order to find new ways of accessing the visual and material properties of early modern knitted stockings and their material and cultural meanings.

## Introduction

Knitting was one of the key areas of technological innovation in early modern Europe.^1^ Although knitting on needles as a textile technique was not new, what was extraordinary about the practice of knitting in this period was the relatively rapid development of knitting from a domestic craft into a prosperous sector of commercial textile production. By the end of the sixteenth century, extensive networks of makers and sellers produced and sold a wide range of knitted garments, from gloves and caps to detachable sleeves and waistcoats, in a broad spectrum of qualities.^2^

**Fig. 1.**
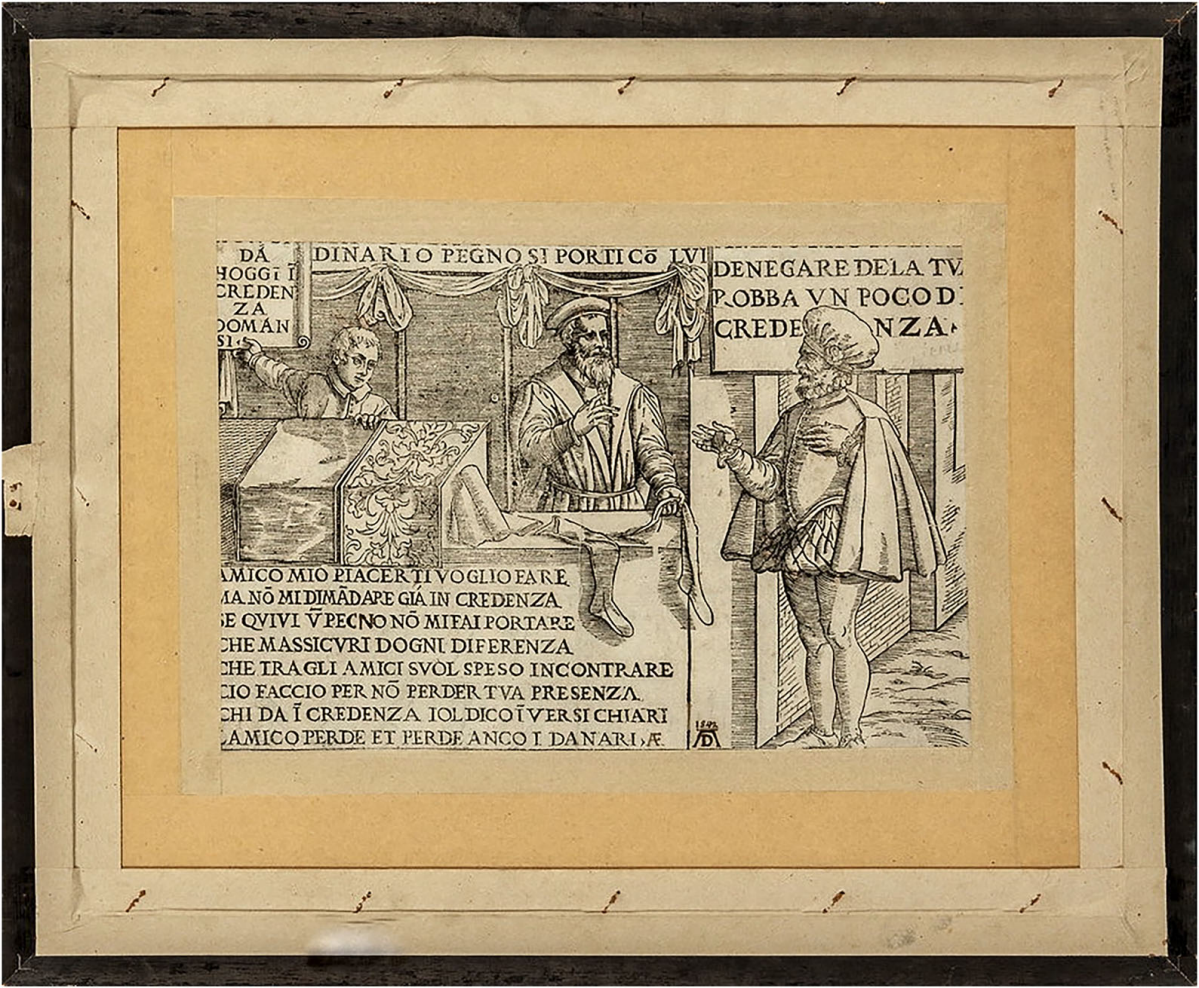
Italian (Venetian?) woodcut, late sixteenth century, showing a stocking at a merchant’s stall. Collection of Luigi Ciompi, no. 2786 (verso). © Photograph by Luigi Ciompi.

One significant development in the knitting industry was the invention of knitted stockings. The growing importance of stockings in the knitting trade is seen in the fact that, by the end of the fifteenth century, there were thousands of professional stocking knitters in Italy.^3^ The industry continued to develop during the sixteenth century and spread rapidly all over Europe.^4^ By the seventeenth century, knitted stockings were available ready-made on the market in varying sizes and shapes (Fig. 1).

**Fig. 2.**
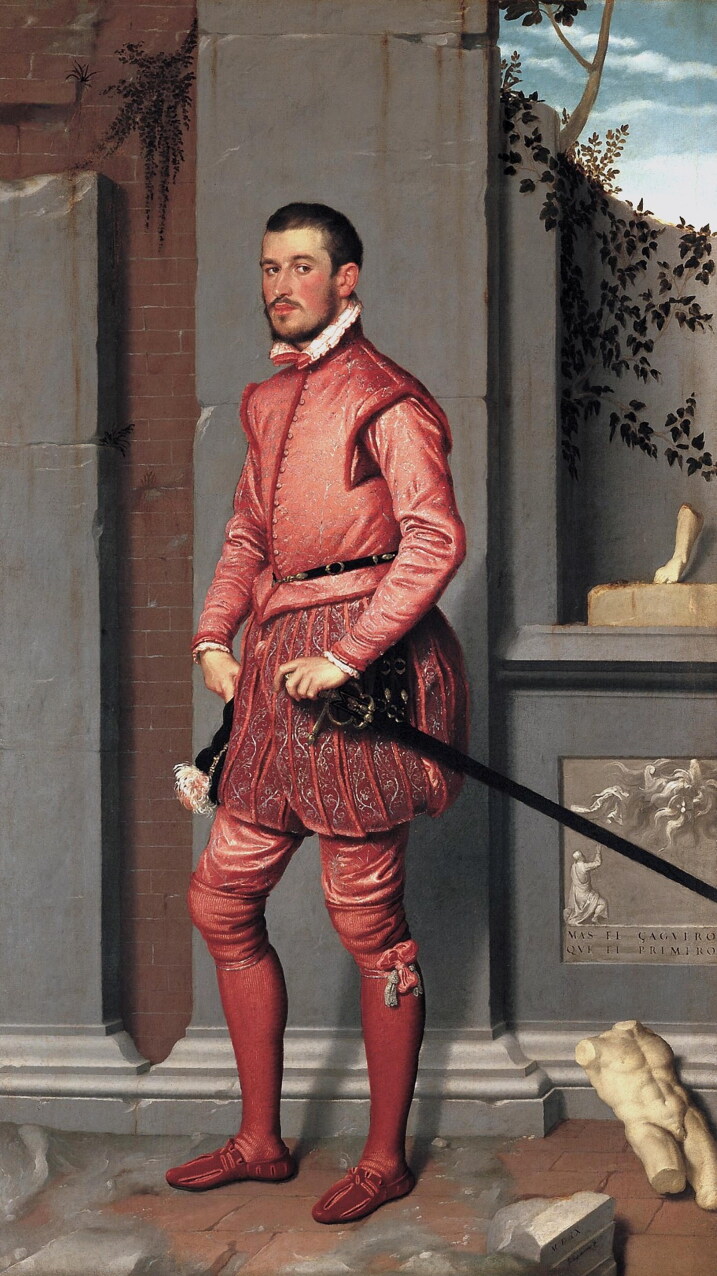
Giovan Battista Moroni, *c*. 1560, Portrait of Gian Gerolamo Grumelli. Fondazione Museo di Palazzo Moroni, Bergamo, Italy. © Photograph by The Picture Art Collection/Alamy Stock Photo.

The invention of knitted stockings not only shaped the knitting industry but also created a European-wide fashion.^5^ Fine, decorative knitted stockings made of silk, produced first in Italy, France, and Spain during the sixteenth century and then also in England in the seventeenth century, became an essential element of the fashionable look.^6^ Visual images and surviving material evidence, such as the extant knitted stockings of Eleonora Toledo, the wife of Cosimo I de’ Medici, show the extent to which fine silk stockings were worn by European men and women of status and rank, not only for warmth, but also for projecting a carefully designed sartorial image that demonstrated awareness of the latest fashions.^7^ Some artists depicted knitted stockings and the tiny stitches with precision. A detail of Moroni’s 1560 portrait of Gian Gerolamo Grumelli, for example, shows how the artist has captured in detail the texture of the young gentleman’s pink knitted stockings (Fig. 2).

Archival records confirm the growing demand for knitted stockings in the early modern period, not just among the privileged rich but also at the lower ranks of society. Family probate inventories and private account books from the sixteenth and seventeenth centuries recorded pairs of knitted stockings in a range of qualities and textures and a wide variety of designs and colours so that even common artisans, such as barbers, shoemakers, and innkeepers, might eventually appear in stockings of silk.^8^ A Milanese wool merchant complained that, by the late seventeenth century, ‘it seems that even people of the lowest grade, carried by their ambition, take shame in using stockings of stamen [worsted] or ordinary wool, using instead those made of *filusello* [spun waste silk] or silk made on frame’.^9^

The growth and importance of the knitted stocking industry from the sixteenth century onwards, especially in Italy but also elsewhere in Europe, has been noted by early modern economic and social historians. They have studied account books, guild records, petitions, trials, notarial acts, insurance policy registers, and probate inventories, revealing how the early modern European stocking industry developed and how it was organised, how raw materials were produced and then supplied to the knitters, and how the production of knitted stockings evolved in different parts of Europe and became partly mechanised with the invention of the stocking frame.^10^ Despite this valuable evidence, our knowledge of the actual knitted stockings—their original visual characteristics, physical form, colours, sensory qualities, and the sense of material comfort—as well as their cultural meaning and early histories of making, remain relatively poorly understood.^11^ What exactly made stockings so appealing for early modern men and women?

This article investigates the making and meaning of early modern knitted stockings. It explores how stockings as fashion accessories became disseminated in sixteenth- and seventeenth-century Europe, examining how and by whom stockings were made; what characterised fine knitted stockings of high fashion in terms of their look, sensory qualities, and the level of skill required for making them; and what made knitted stockings fashionable. The exploration is based on a new methodology that combines early modern visual and archival records, including a dataset of over 80,000 items of clothing, accessories, and decorative embellishments collected by the Refashioning the Renaissance Project from post-mortem inventories from Siena, Florence, and Venice, between 1550 and 1650, with reconstruction of early modern knitted stockings.^12^ This blend of traditional historical sources with historical reconstruction allows us to explore not only the meanings, uses, and dissemination of knitted stockings through visual or written records, it also enables us to examine how the material properties of early modern knitted stockings contributed to their appeal, economic value, and cultural meanings.

**Fig. 3.**
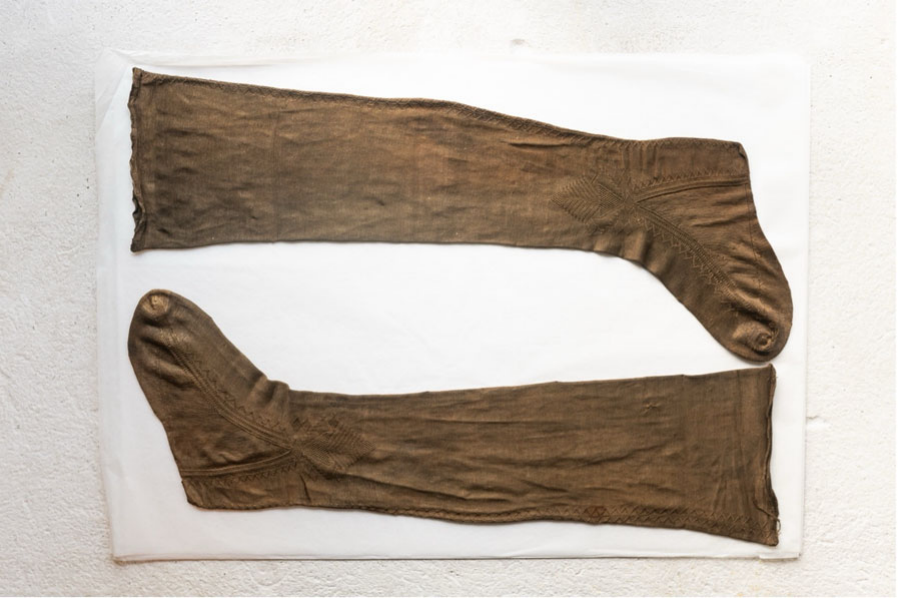
Silk stockings, mid-seventeenth century, Turku Cathedral Museum. Catalogue number 1364d (above) and 1755b (below). The reconstruction was made from 1364d. Photograph by Jussi Virkkumaa. © Refashioning the Renaissance.

**Fig. 4.**
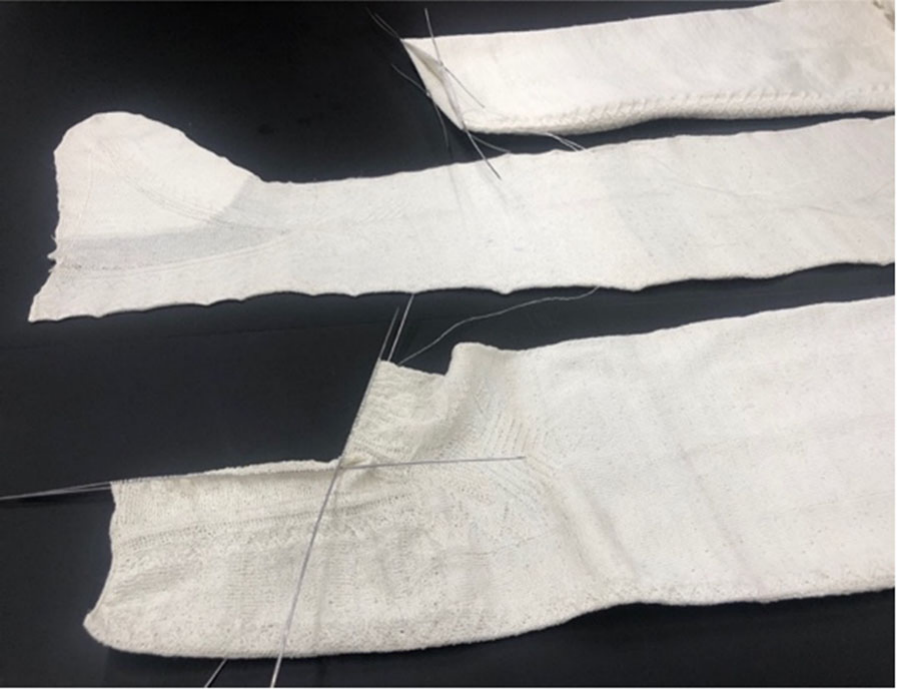
Reconstructions of the silk stocking 1364d in progress. Photograph by Piia Lempiäinen. ©Refashioning the Renaissance.

The reconstructions in this article are the outcome of a research experiment, organised as part of the wider research project on Renaissance clothing and fashion (European Research Council-Refashioning the Renaissance, 2016–2022, Aalto University, Helsinki), in which the project’s academic researchers and thirty-five local volunteer participants reconstructed early modern knitted stockings, including an extant seventeenth-century fine hand-knitted silk stocking from Turku in Finland (Figs 3 and 4).^13^ Previous experimental archaeology projects on early modern knitting, such as the recent Texel stocking project in Leiden, which used close to 100 volunteers to reconstruct seventeenth-century silk stockings that were recovered in 2014 from a shipwreck near the island of Texel in the north coast of the Netherlands, provided valuable models for how to gain access to the production and construction of historical textile objects using ‘citizen science’.^14^ Although such methods where researchers or audiences learn or know through re-enacting or replicating have obtained a growing importance in experimental archaeology, reconstruction is a relatively new approach in the academic practice of material culture and cultural studies of dress.^15^ It still needs to be established, as Hilary Davidson has pointed out, what we can learn from experimental archaeology to suggest that ‘experimental history’ is an equally valid concept.^16^

This article uses reconstruction and close analysis of surviving textile objects in conversation with a range of other historical sources and methods, in order to fill the gaps of knowledge in cultural research of dress about the craft skills and the value of labour, material sourcing, and knitting tools, the look and colours of stockings, and the experience of making and wearing early modern knitted stockings. By examining a large archival dataset and bringing some of the knitted stockings to ‘life’, it shows the ways in which material reconstruction can help us explore how the numerous fine, colourful stockings described in documents or painted in pictures were actually made, what level of skill and sophistication was required to make different types of stockings, and what qualities made knitted stockings so desirable in the sixteenth and seventeenth centuries.

## The Dissemination of and Fashion for Knitted Stockings

**Fig. 5.**
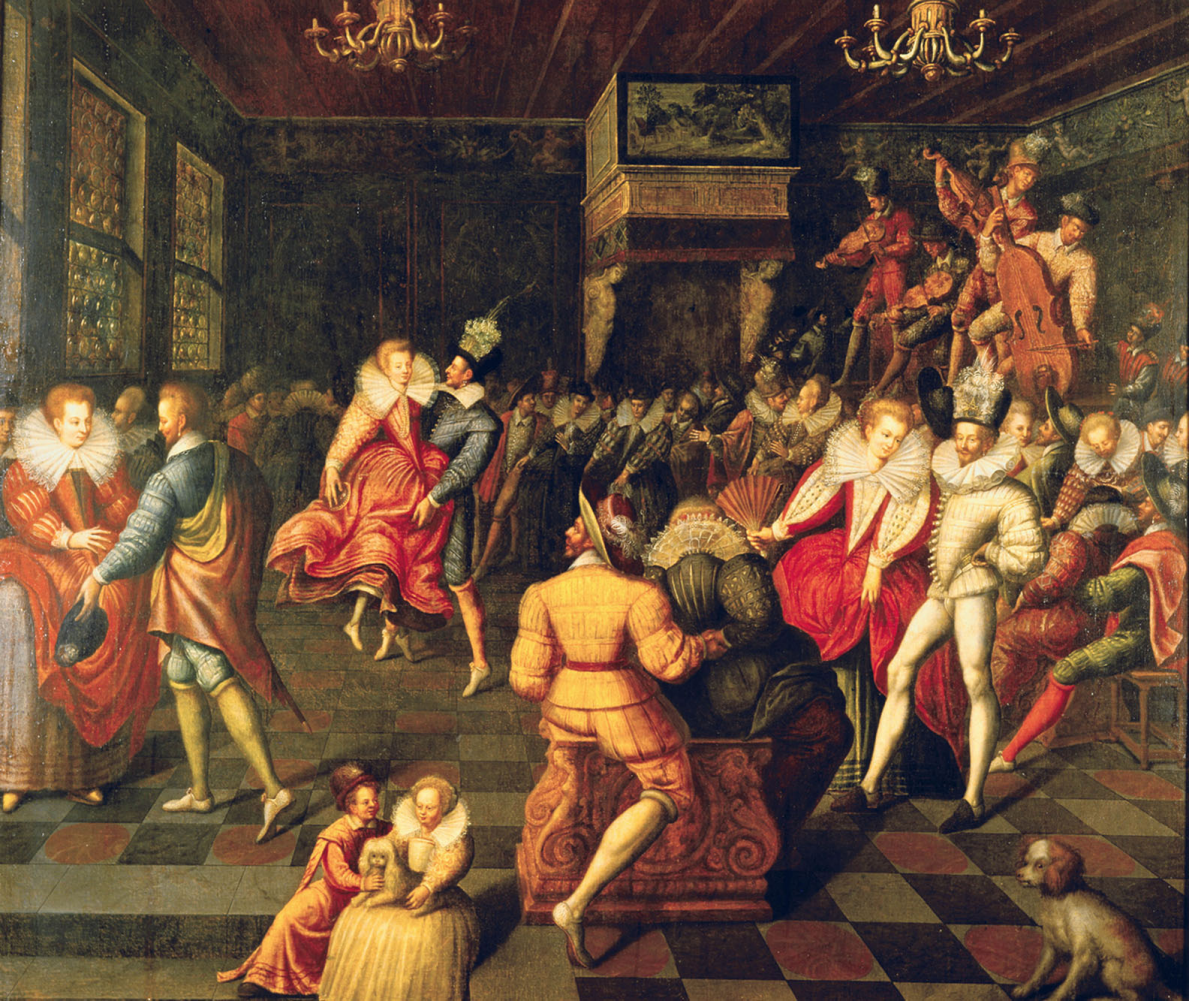
*Ball at the Court of Valois*, French School, sixteenth century. Museum of Fine Arts at Rennes. © Photograph by World History Archive/Alamy Stock Photo.

**Fig. 6.**
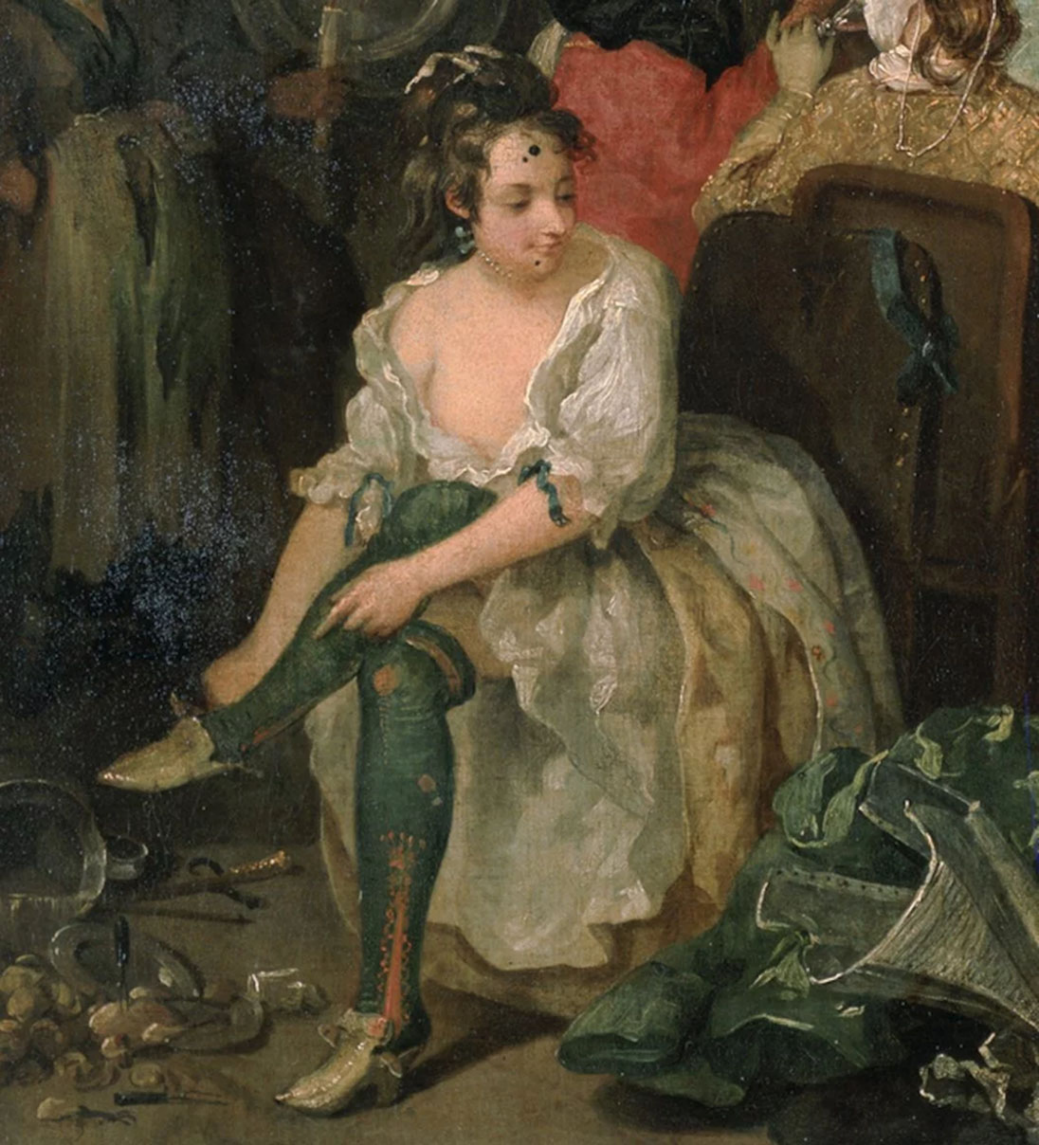
William Hogarth, Detail from *The Tavern Scene (A Rake’s Progress)*, between 1732 and 1735. Oil on canvas. London: Sir John Soane’s Museum 1868,0822.1530. © Photograph from Wikimedia Commons, public domain.

Visual and written evidence shows that knitted stockings became an indispensable fashion accessory in early modern Europe. According to Cesare Vecellio, the sixteenth-century writer who compiled the first printed costume encyclopaedia, fashionable stockings knitted on needles and made of the ‘finest silk’ were widely worn in his hometown of Venice by the second half of the sixteenth century, especially by ‘gentlemen and merchants and by noble and rich youths’.^17^ Knitted stockings appealed to men and women because they were colourful, extravagant, and new.^18^ Fine stockings were increasingly on offer in fashion centres, such as Paris and Italy, in a variety of styles and ranging from plain stockings to those decorated with embroidery, diagonal bands, or patterned clocks (decorated sections on the sides of the ankle parts of the stockings).^19^ These varied in colour, from black stockings favoured for formal occasions to brightly dyed reds, yellows, and greens that were worn in more informal gatherings, balls, and during festivities. By the mid-seventeenth century, stockings could be bought in as many as fifty different shades.^20^ The variety of colours has been captured in contemporary depictions, such as, for example, in the sixteenth-century image depicting the ball at the Valois court, where men appear in stockings in shades of yellow, white, green, and red (Fig. 5). Although women’s stockings were rarely visible in visual depictions because they were hidden by their skirts, early modern documents such as notarial inventories and mercantile records demonstrate that women wore bright and decorative knitted stockings too, sometimes even more daring in colour than those worn by men.^21^ A slightly later image from the eighteenth century by William Hogarth, showing a women taking off her shoe in a tavern scene, demonstrates the fine detail and decorative appearance of stockings worn by women (Fig. 6).

Knitted stockings were desired not only for their novelty and appearance but also because they provided a better fit and greater comfort than the traditional cloth hose, which were cut and sewn by tailors. Knitted fabric was elastic and even a tight-fitting knitted stocking could be put on and taken off the leg with ease in comparison with stiff stockings of cloth.^22^ An anecdote from the ‘Life of the painter Cristofano Gherardi’ in Giorgio Vasari’s *Lives* (1568) illustrates how laborious it was to take off a traditional tight-fitting cloth stocking. The story tells how one painter named Cristofano, unable to take his stocking off after an evening’s party, had gone to sleep with one leg bare and the other covered with the stocking. Two servants were apparently needed to draw the remaining stocking off. With one servant holding Cristofano’s leg and the other pulling at the stocking, the servants managed to get the stocking off, while the painter himself, according to Vasari, lay on the bed ‘cursing the clothes … and him who invented such fashions that … kept men bound in chains like slaves’.^23^

The close fit of the stockings added to their appeal especially for men because they emphasised the shape of their legs. At first, knitted male stockings were long tights covering the entire leg and stitched to the breeches. During the second half of the sixteenth century and first half of the seventeenth century, however, knitted stockings were usually separated from the breeches and worn with elaborate garters.^24^ Combined with decorative silk and lace garters and worn with high-heeled shoes, tight-fitting stockings drew the attention to the male legs, regarded as one of the most beautiful and desired parts of men’s bodies.

**Fig. 7.**
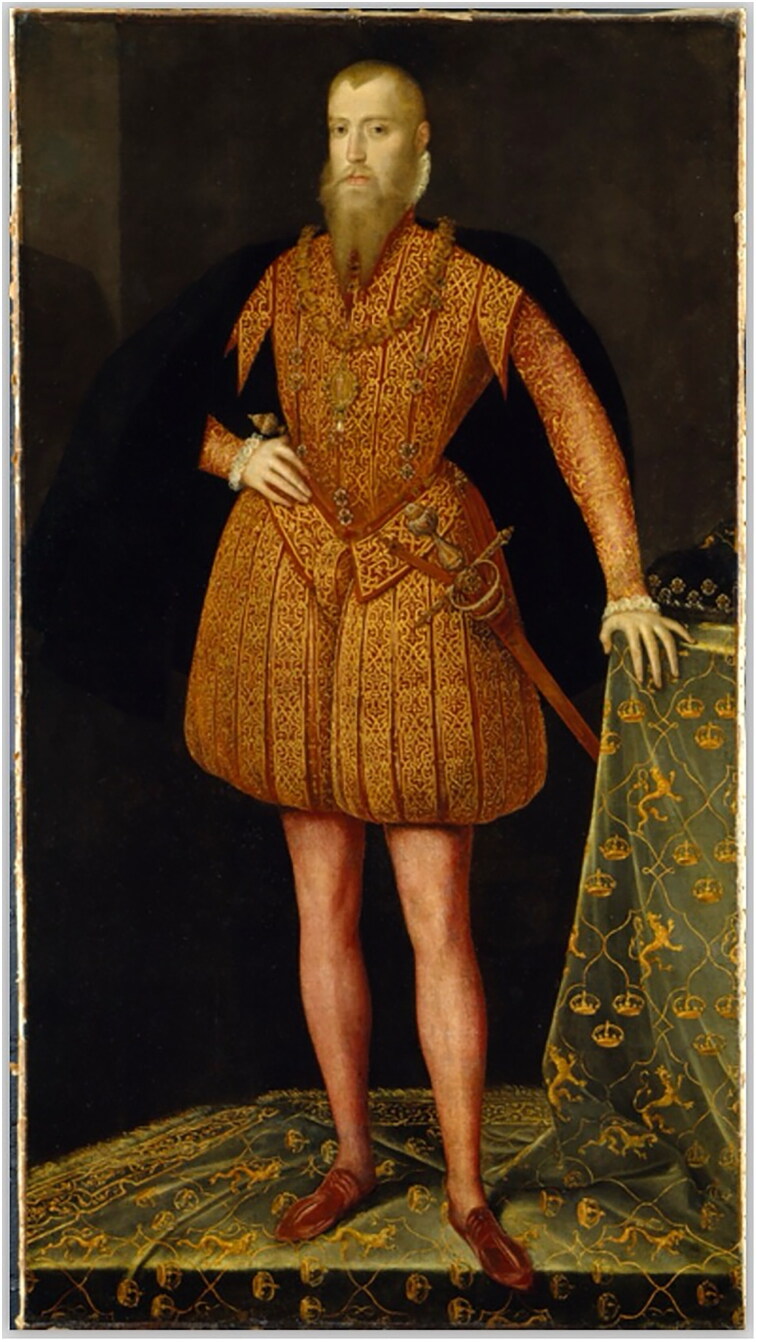
Erik XIV of Sweden, attributed to Steven van der Meulen, 1561. National Portrait Gallery. © Photography by ART Collection/Alamy Stock Photo.

The fashion for knitted stockings was not confined to the most important centres of production, such as Italy, England, or France. The demand spread quickly across most of Europe, reaching as far as Scandinavia.^25^ King of Sweden, Eric XIV, for example, seems to appear in his 1561 portrait in tight-fitting stockings of red silk (Fig. 7).^26^ Although knitted silk stockings in peripheral countries such as Sweden were so rare that until the end of the sixteenth century, they were worn almost exclusively at royal courts, account books and customs records show that private families increasingly were able to acquire ready-made knitted stockings from abroad. Customs records from Turku show that, in 1585, three pairs of silk stockings—one in red, another in flesh-colour, and the third in purple—were imported from Lübeck to the Swedish province of Finland for local affluent families. Fine knitted stockings such as these were so precious that they were ordered only for special occasions. For example, in 1615, the governor Hans Stålhandske ordered from a merchant in Vyborg one pair of silk stockings for his wedding.^27^

The wide geographical dissemination of fine knitted stockings, visible in written records, also is evidenced by surviving material evidence. A mid-seventeenth-century hand-knitted silk stocking, conserved at the Turku Cathedral Museum in Finland, confirms that by this date silk stockings had become a feature of dress also among families at the more peripheral areas of Europe. The stocking was found in the burial coffin of Elizabeth Bure, the wife of the Vice President of the Court of Appeals (Fig. 3).^28^ Although it is not known where the stocking was originally made, surviving seventeenth-century examples suggest that it was similar in style and quality to some of the finest knitted stockings produced in England in this period.^29^

Visual and written records demonstrate that the fashion for knitted stockings, furthermore, was not limited to the wealthy elites. The Refashioning the Renaissance Project’s dataset, based on 448 household inventories of artisans and small shopkeepers from Siena, Florence, and Venice between 1550 and 1650, included in total 2,361 pairs of stockings, making it on average approximately five pairs of stockings per household.^30^ The materials mentioned in records indicate that, from the late sixteenth century onwards, tailored cloth hose made of heavy silks (*velluto*) or wool cloth (*panno*, *scarlatto*, *saia*, *rascia*) were increasingly complemented and largely replaced after the 1590s by stockings knitted of silk (*seta*, *filusello*, *filaticcio*, *bavella*) or wool yarn (*lana*).^31^ Approximately ten per cent of the stockings were made of silk. About one sixth of these were knitted using lower grade spun silk thread called *filusello* or *bavella*, made of short waste silk fibres or damaged cocoons.^32^

Although stockings of spun silk were not comparable in quality to the finest stockings worn by the wealthy elites, made using slightly twisted thin long silk filaments, knitted stockings worn by the ordinary artisanal population such as barbers, bakers, or shoemakers seem to have been treasured objects of novelty and fashion. Stockings listed in their post-mortem inventories were often dyed in bright colours and a variety of shades that corresponded with the latest colour fashions of the seventeenth century, such as medium blue (*turchino*), dove grey (*colombino*), silver (*argentino*), aquamarine (*aquamare*), brown (*roan*), golden yellow (*doreto*), milky white (*latado*), and black (*nero*). A Venetian papermaker, Andrea, for example, who died in Venice in 1611, owned a dashing pair of stockings of knitted silk *bavella*, dyed golden yellow (*dorete*).^33^ The look of the stockings could be enriched with decorative silk garters. For example, one Sienese innkeeper, Francesco Pallino, owned a pair of golden yellow silk stockings with ‘a measure of taffeta for garters’, while the Sienese leather dealer Vincenzo de Rosis who passed away in 1639 had a pair of stocking made of half silk (*teletta*) and decorated with ‘Neapolitan ribbons’.^34^ Combined with decorative garters of silk and lace, knitted stockings could provide an important way for sixteenth- and seventeenth-century artisans and burghers below the high-ranking elites to connect with the latest fashions.

Fine, hand-knitted silk stockings, such as the silk stocking from Turku, were expensive fashion accessories. Silk stockings imported from Naples—then an important centre of production for silk stockings—could cost as much as seven *scudi* a pair, a sum close to two months’ salary of a master craftsman.^35^ Decorative stockings, using the same knitting pattern, were also made of fine wool.^36^ Wool stockings were used by all social classes, often as under-stockings, but when knit of a fine worsted wool, they provided a visually attractive alternative to knitted silk stockings. Although wool was generally cheaper than silk, according to Philip Stubbs, the author of *The Anatomie of Abuses* (England, 1583), some wool stockings were so fine that they were comparable in price with silk.^37^

## The Makers of Stockings

Stockings were knitted by hand until William Lee, the son of a yeoman farmer of substantial means in Calverton, Sherwood Forest, Nottinghamshire, invented the first stocking knitting-frame in 1589.^38^ The knitting-frame, in which the machine replicated the actions of hand-knitting by moving a length of thread into the loop at the end of the needle, then applying pressure to close the needle and finally sliding the existing length of fabric over the top, allowed knitters to produce stockings three or even six times faster than making stockings by hand.^39^ Although the invention and diffusion of the stocking knitting-frame sped up production and gradually transformed the industry for knitted stockings, especially in silk, the technical challenges associated with the frame, and the investment and professional skill required to set it up and operate the delicate mechanism, ensured that most stockings at the end of the sixteenth century and early seventeenth century were still produced by hand, and hand-knitting continued to prosper until the nineteenth century.^40^

**Fig. 8.**
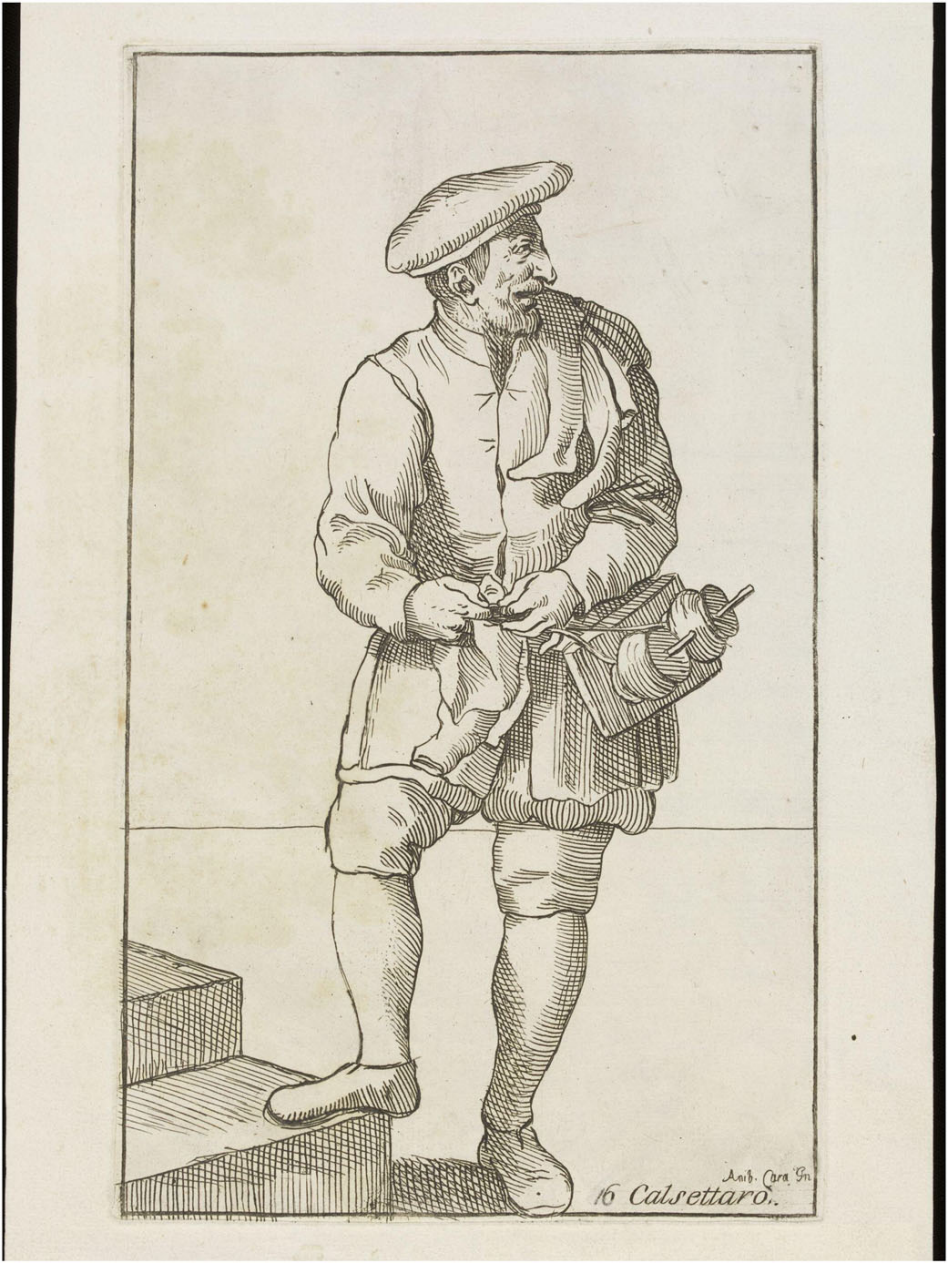
An image of a ‘hosier’ from *Le Arti di Bologna*, made by Simon Guillain after Annibale Carracci, etching, first published in 1646. © Photograph from Wikimedia Commons, public domain.

Scholars writing about the early modern period have argued that the relative simplicity associated with hand-knitting made knitting wool socks and stockings a suitable activity for all. Thousands of individuals without any professional training, including women, children, and the elderly, appear to have been employed in the knitting industry by the end of the fifteenth century.^41^ The repetitive nature of the craft meant that knitting could also be easily carried out by many as a side activity at home to gain supplementary income. Household inventories of shopkeepers and artisans sometimes listed considerable numbers of stockings, suggesting that these men or their wives made knitted stockings for the market. The inventory of one Venetian dairy-product seller named Francesco and his wife, for example, included a stock of ninety-six stockings in his home, while the inventory of the Sienese spinner Claudio Borselli, mentioned in total forty pairs of stockings.^42^ Knitting was also widely practised by farmers and shepherds who could integrate it with their agricultural work. A document concerning one Andrea Fuoghi, for example, reveals that when he was invited by a Vescovile notary to declare his occupation, he responded, ‘I do work of knitting when I don’t have the occasion to go out and work at the land’.^43^ An image of a ‘hosier’ from *Le Arti di Bologna*, made by Simon Guillain after Annibale Carracci (first published in 1646), demonstrates how knitting could be carried out while working or walking outdoors (Fig. 8).

Although Italian knitting for the commercial market was carefully controlled by merchants and the guilds, archival records, such as petitions submitted for the Florentine wool guild Arte della Lana, show that knitting was widely practised illegally outside the guild system.^44^ The Florentine silk dyer Piero di Jacopo, for example, was charged ten *scudi* in 1610 for knitting and dyeing a pair of red stockings of high-quality wool (*stame*) that he had not declared.^45^ Also, peasants and soldiers often were charged with either making stockings without appropriate licences or failing to pay the duties set by the guilds. In 1610, a shepherd named Raffaello di Giovanni, for example, was charged by the wool guild for making and hiding two pairs of knitted socks and one pair that was still in progress. He was consequently thrown in jail and his horse and sheep confiscated.^46^ The soldier Pietro d’Antonio from the Fortezza di San Martino di Mugello was also condemned by the wool guild for having ‘knitted stockings and socks and other works of ordinary local wool’.^47^ The penalties for the illegal practice of knitting could be severe. A certain Pierino di Ulivio from Castagno, for example, was sentenced to the galleys for a period of five years, after he had been caught smuggling through customs in Florence ‘a white knitted wool sock’, among other goods he had not declared to the officials.^48^

Although most stockings knitted by peasants or children without specific training were probably relatively ordinary socks and stockings of wool, silk stockings were also produced by women at home. Since thin silk material was delicate and expensive and the construction was often relatively complex, knitting fine silk stockings required a high level of skill and care. A 1716 document recording mercantile activities in Milan expressed great concern for the limited care with which some early modern knitters treated the silk stockings they made, claiming that knitting women often ‘spoiled the colours and handled the stockings with dirty hands’.^49^

However, the ways in which silk stockings were made, the level of skill required to make them compared to knitting ordinary wool socks and stockings, and how the making process contributed to their value is not well known. The early history of making knitted stockings is relatively unfamiliar in the cultural history of dress because instructions or knitting patterns do not survive from the period.^50^ In the absence of written historical record, the examination and reconstruction of surviving material evidence provides an essential method to investigate how fine stockings in this period were constructed, what materials and tools were used to make them, and what level of skill was required in making the fine knitted fabric and decorative patterns of fine stockings of fashion.

## The Construction of Knitted Stockings

The surviving seventeenth-century silk stocking from the Turku Cathedral Museum offers a good case study for the examination and reconstruction of an early modern fine silk stocking of fashion, because it is in exceptionally good condition with no visible holes and only a few loose threads (Fig. 3). It provides an example of a fine decorated silk stocking worn by the elites in the period. In common with many knitted stockings of the period, the design imitated the cut and construction of the traditional cloth stocking, which had a seam at the back. There is a false zigzag seam at the back, a decorated clock on the side of the ankle part of the sock, and a zigzag decoration at the edges of the heel gusset, all made with purl stitches.^51^

**Fig. 9.**
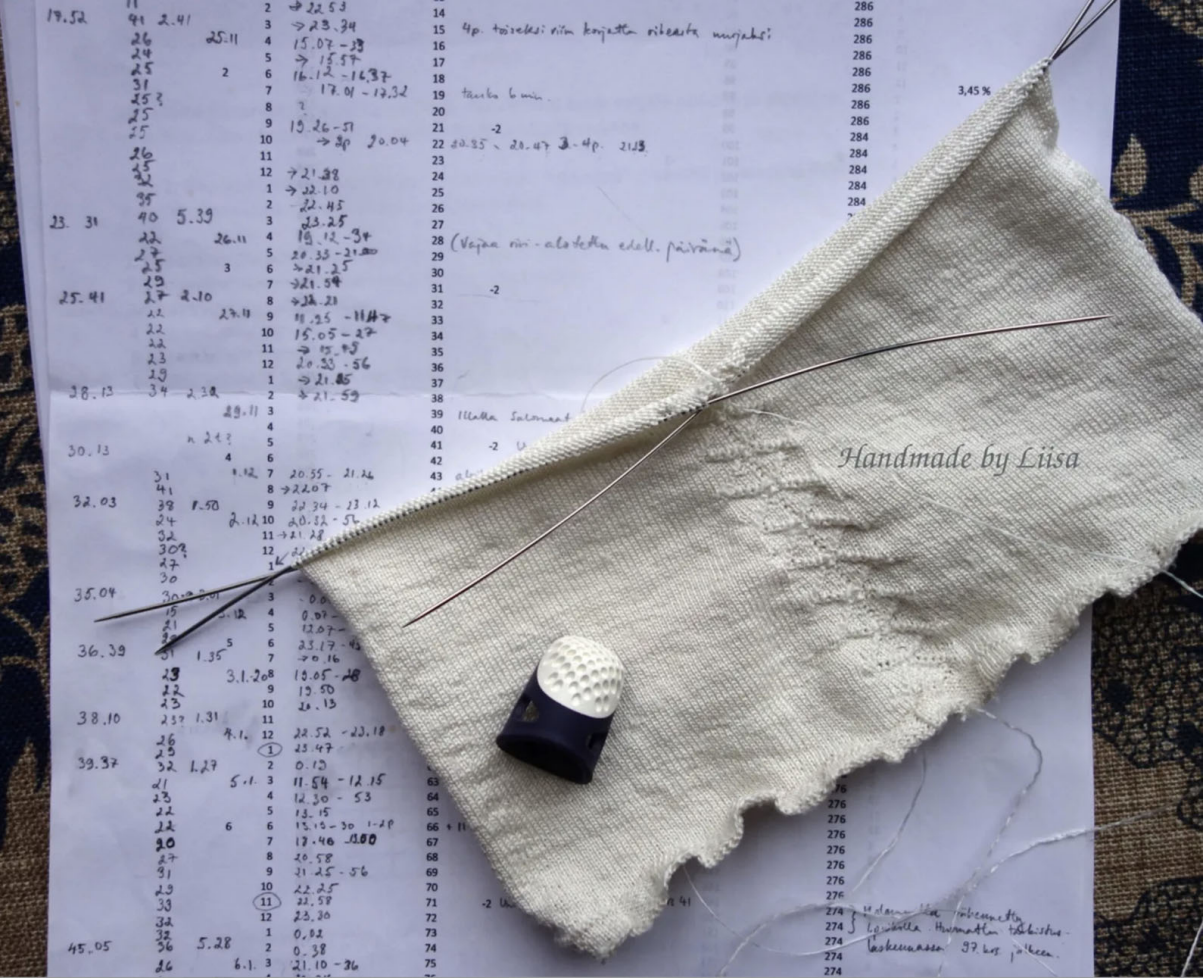
Knitting pattern for the Turku silk stocking 1364d. © Photograph by Liisa Kylmänen.

The Turku stocking was reconstructed at Aalto University in 2019–2022 by the Refashioning the Renaissance Project and thirty-five volunteers who joined the project following our call on our website and social media in 2019 to participate in a historical research experiment. All our knitters were skilled non-professional female knitters between thirty and seventy years of age. The aim of the experiment, designed by the Refashioning project, was to replicate the fine stocking as accurately as possible, in order to study how a complex early modern stocking was made, what the experience of knitting an early modern stocking was, and what made such a stocking so desirable and fashionable in the early modern period. A close examination of the original stocking provided a starting point for the reconstruction (Fig. 3). The length of the stocking, measured along the false zigzag back seam from top to the bottom of the back heel, was 61 cm and it contained in total over 900 courses (or rounds) of tiny stitches of thin silk yarn, with as many as 120 courses by 80 loops per 10 cm.^52^ The stocking was knit in the round from the top down in stocking stitch, with all decorative stitches made with purl stitches. Based on the technical analysis, the most experienced knitters from the group of volunteers counted every stitch of the stocking and drafted a pattern for the reconstruction (Fig. 9).^53^

The first step of the reconstruction was to investigate from what type of silk material the stocking was made and to purchase a yarn that was consistent with the historical stocking. A fibre analysis of a small sample taken from near the opening of the original stocking, analysed with Scanning Electron Microscopy (SEM) and Energy Dispersive X-Ray Analysis (EDX) at Aalto University Nanomicroscopy lab, indicated that the surviving silk stocking from Turku was made of Bombyx mori silk, the finest silk yarn available at that date. The silk thread of the original stocking was made of long and very fine, slightly twisted silk filaments.^54^

**Figs 10–11.**
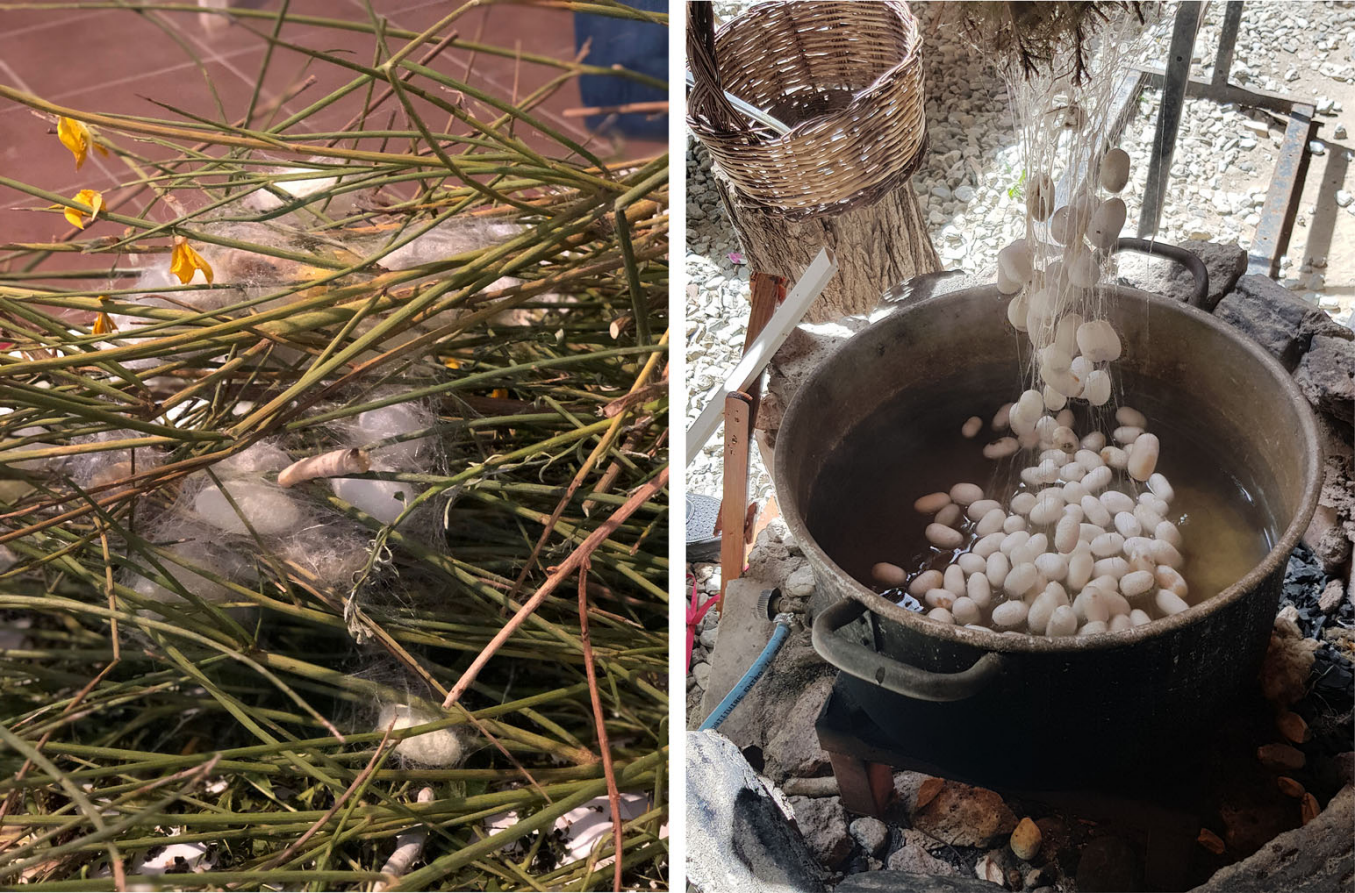
Silk worms ‘spinning’ the cocoons and silk preparation, Nido di Seta, 2019. © Photograph by Refashioning the Renaissance.

**Figs 12–13.**
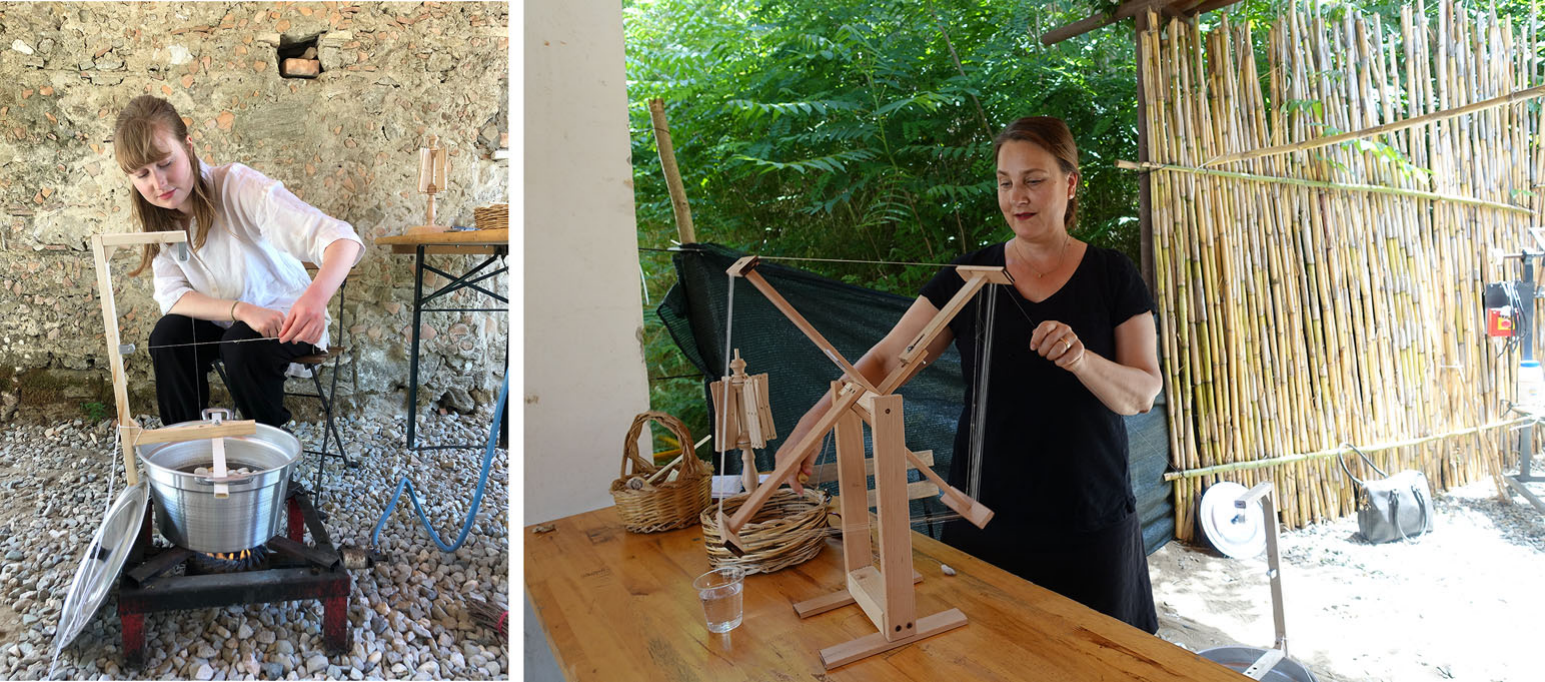
Silk reeling set up and the process of reeling. Nido di Seta. © Photograph by Refashioning the Renaissance.

Bombyx mori—a domesticated silk moth breed—originated in China and was widely cultivated in Europe by the seventeenth century.^55^ This type of silk, however, is no longer commercially available because the industry was almost destroyed between the eighteenth and nineteenth centuries, due to the Pebrina epidemic that made infected silkworms unable to produce silk thread. Fortunately, a small co-operative, Nido di Seta, had recently established a small silk farm in a centuries-old mulberry tree forest near the village of San Floro in Calabria, Italy in an attempt to produce Bombyx mori silk in the traditional local way.^56^ A workshop organised by Nido di Seta farm enabled us to examine how precious silk thread, such as that used in the Turku stocking, was made (Figs 10–13).^57^

**Fig. 14.**
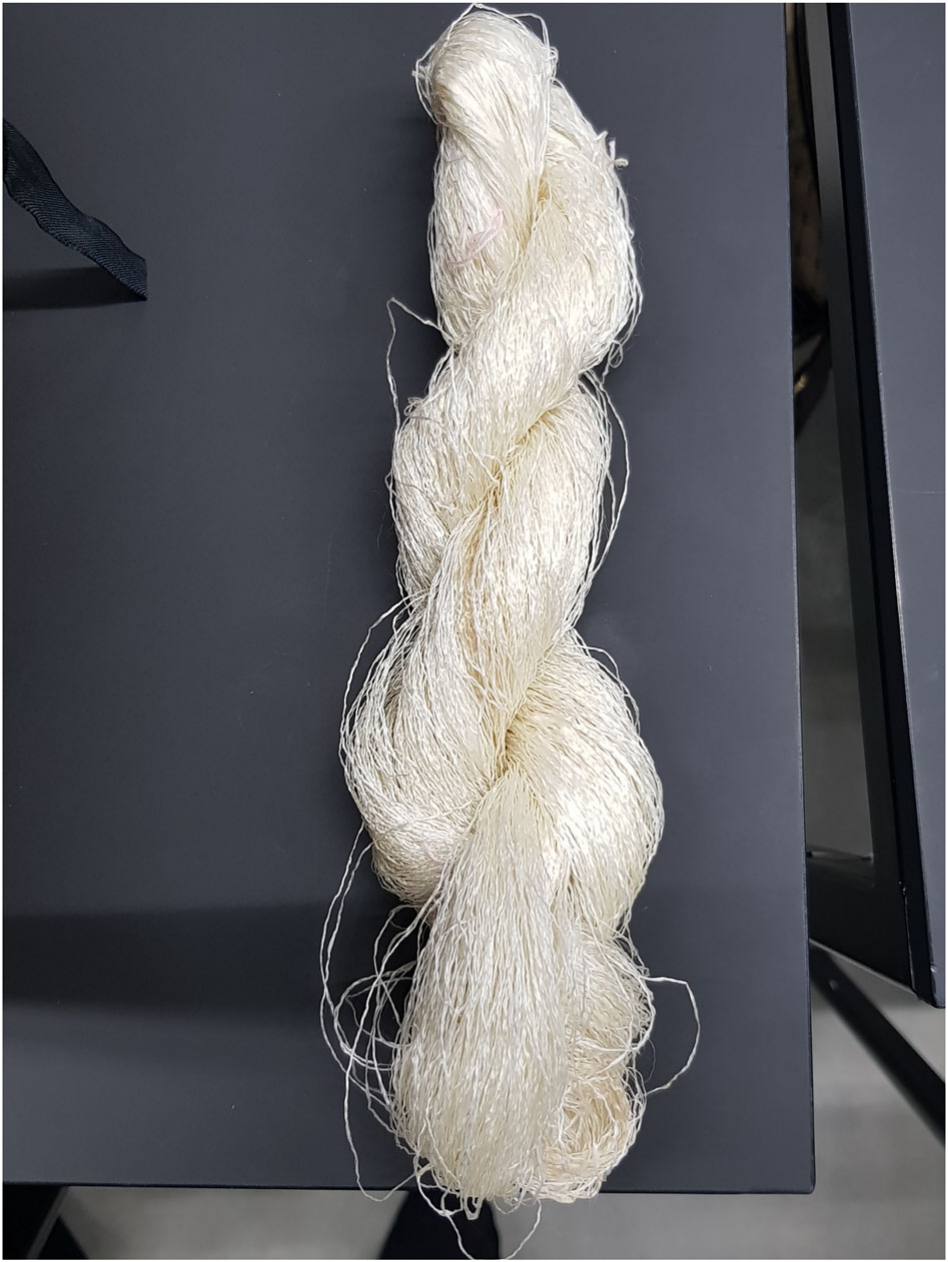
Silk yarn from Nido di Seta. © Photograph by Refashioning the Renaissance.

**Fig. 15.**
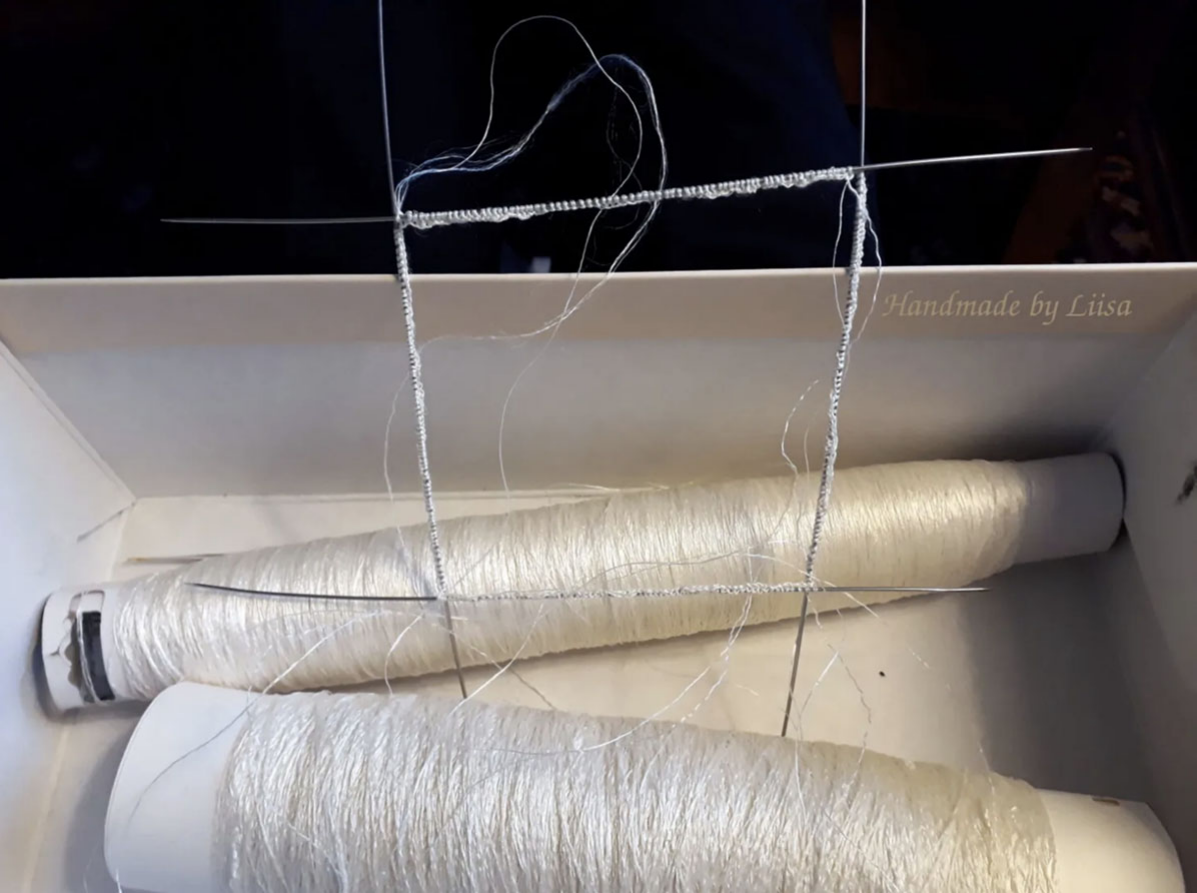
Knitting needles and hand-reeled Bombyx mori silk from Nido di Seta, used for the Turku silk stocking reconstruction. © Photograph by Liisa Kylmänen.

The quality of the raw silk in the seventeenth century was one of the most critical factors that determined the quality of the stockings; the silk yarn had to be even and well processed. Writing on the importance of the quality, an eighteenth-century treatise by M. March, published in London, explained how raw silk of optimal quality is prepared, first by using the correct number of cocoons and then by skilfully doubling the silk. He wrote:

Silk in its first state is curiously spun in the shape and size of a walnut which is called *cacoon* [sic], several of which are put into warm water and run together on a reel so as to make thread of raw silk; after it is dry, the skeins are wound, doubled and thrown … The first spinning or outside of the *cacoon* is the thickest silk and the inside or last spun is the finest, which shows how necessary it is the *cacoons* should be carefully doubled, for when the silk is run half off it requires an additional thread or *cacoon* and sometimes more to be added to keep the silk even; yet this is too frequently neglected, which in some degree accounts for the thick and fine parts in silk.^58^

Our collaboration with Nido di Seta allowed us to obtain hand-reeled silk that corresponded as closely as possible to the authentic seventeenth-century silk used in the Turku stocking.^59^ The volunteers knitted several small swatches, using different sets of Nido di Seta hand-spun yarn, to find out which yarn resulted in a similar gauge and appearance as the original stockings. After several test batches, we decided that the best result and similar gauge could be achieved using a silk yarn that had been made of long silk filaments using 150 silk cocoons from the 2019 harvest and given a slight twist to reinforce the thread (Figs 10–13).^60^ Although the silk thread made by hand at Nido di Seta corresponded relatively well with the thickness of the original seventeenth-century silk yarn of the Turku stocking, producing uniform high-quality silk thread by hand proved to be challenging and required a high level of skill.^61^ The finished thread was not entirely even and, in addition, some of the silk strands had glued together during the drying process due to the exceptional humidity in Calabria during the summer of 2019. This was because silk contains glue-like sericin before it is washed off (Fig. 14).

**Fig. 16.**
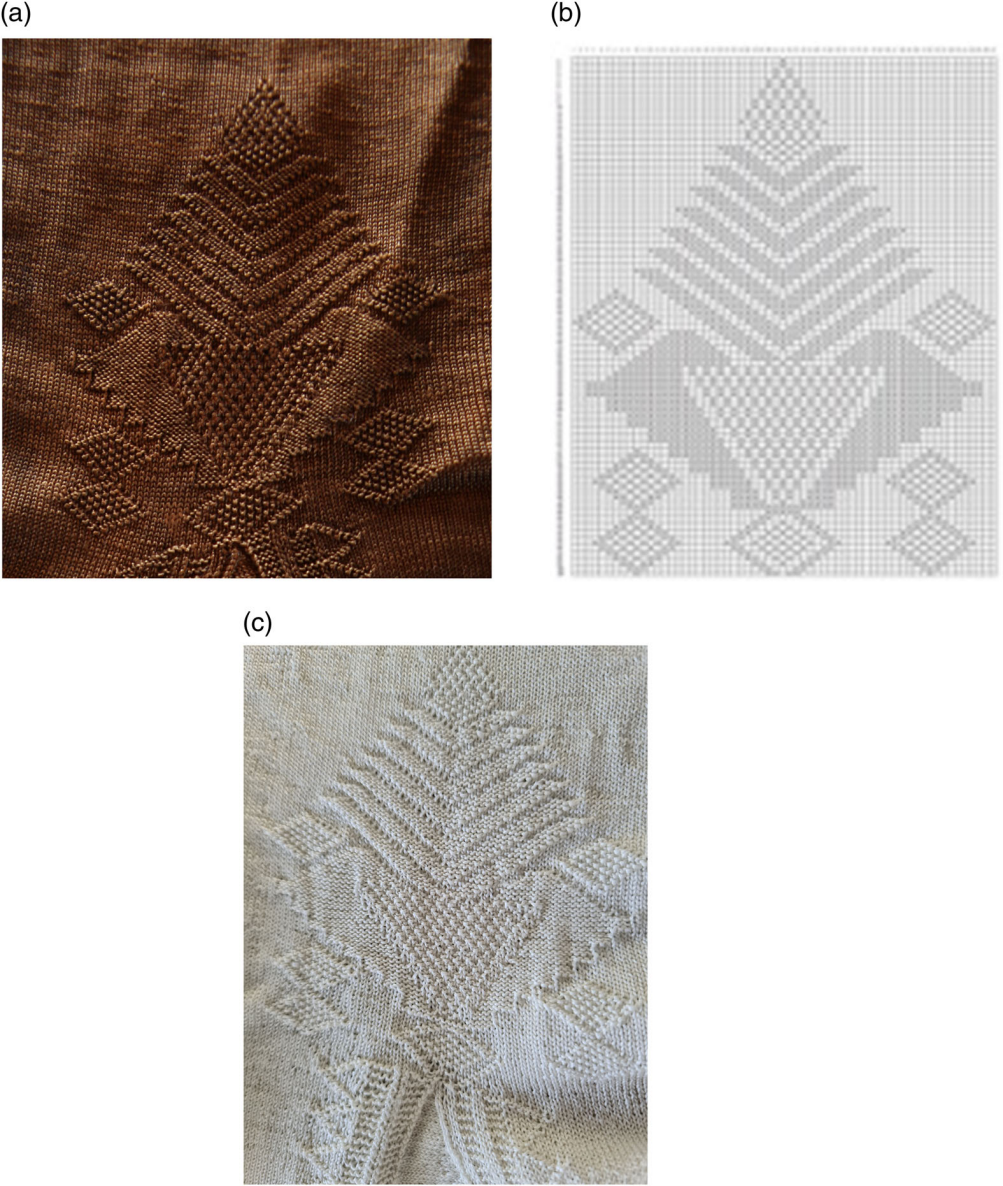
Clock pattern of the Turku silk stockings 1364d: a) original stocking; b) knitting pattern; c) reconstruction. © Photograph by Refashioning the Renaissance.

In order to test the knitting process in fine wool using the same pattern and to compare the look and sensory qualities, we also selected a woollen alternative for the silk. A sixteenth-century writer familiar with the techniques of hand-knitting stated that fine, long-staple wool with flexibility ‘enabled the knitters by hand in woollen goods to keep their superiority’.^62^ The wool for the reconstructions of the seventeenth-century Turku stocking was selected based on test swatches made by the volunteer knitters, and we chose two types of commercially available fine undyed worsted wool yarns that were not superwash treated, today most often used in weaving.^63^

## Knitting Early Modern Stockings

The knitting stage of the reconstruction started by finding historically appropriate needles. Early modern evidence suggests that fine silk stockings were made ‘using only four thin iron rods’.^64^ Although knitting needles from the period have not survived, they are mentioned in early modern archival records and sometimes depicted in paintings, such as in the famous image by Master Bertram (*c*. 1370), where the Madonna is knitting a pink crimson shirt with a set of needles.^65^ Good knitting needles in the sixteenth and seventeenth centuries were described as ‘long, even and firm … and also a little coarse’ and, according to the sixteenth-century ‘Song of Knitters’ (*Canto degli agucchiatori*) by Giovambattista Gelli, the needle also had to be ‘smooth so that it could move faster’.^66^ We interpreted this that the needles need to be a little ‘sticky’ so that they grip the yarn a little, especially when knitting silk, but smooth enough to not snag the fine yarn.

The test swatches made during the training sessions by the project’s knitters, using different yarns and needles, indicated that to replicate the Turku stocking and arrive at the stitch count of the original, the knitting had to be done using very fine needles, between 0.7–1 mm. For the reconstruction, we selected a set of five double-pointed steel needles, with a length of 10 cm, which were characterised by a very slim point and very good sliding properties (Fig. 15).^67^ Knitting fine yarn in both wool and silk with thin needles such as these made the work challenging. One of the volunteer knitters reported:

**Fig. 17.**
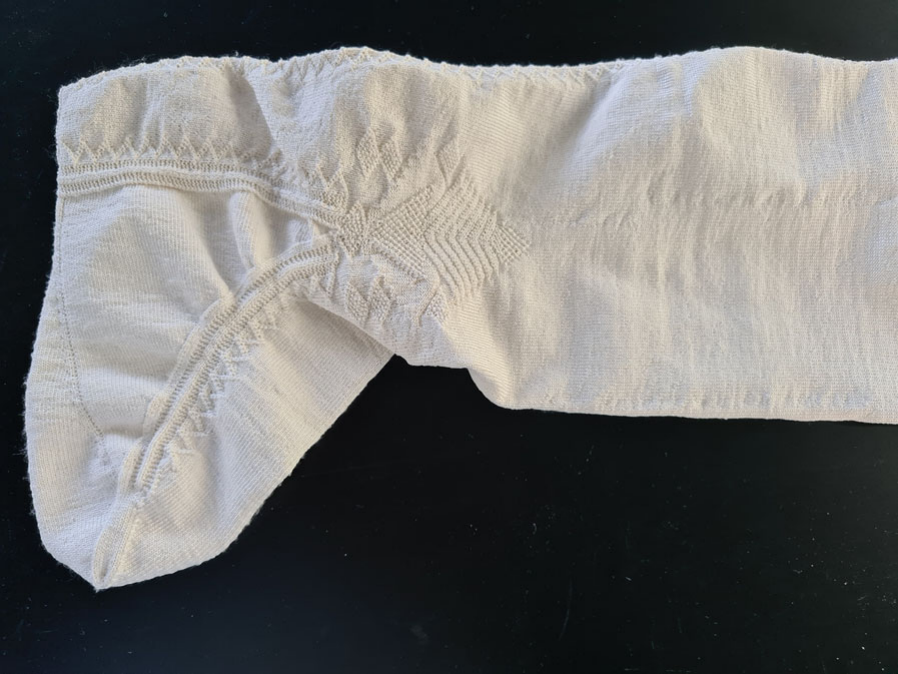
Degummed silk stocking reconstruction. © Photograph by Refashioning the Renaissance.

**Figs 18–19.**
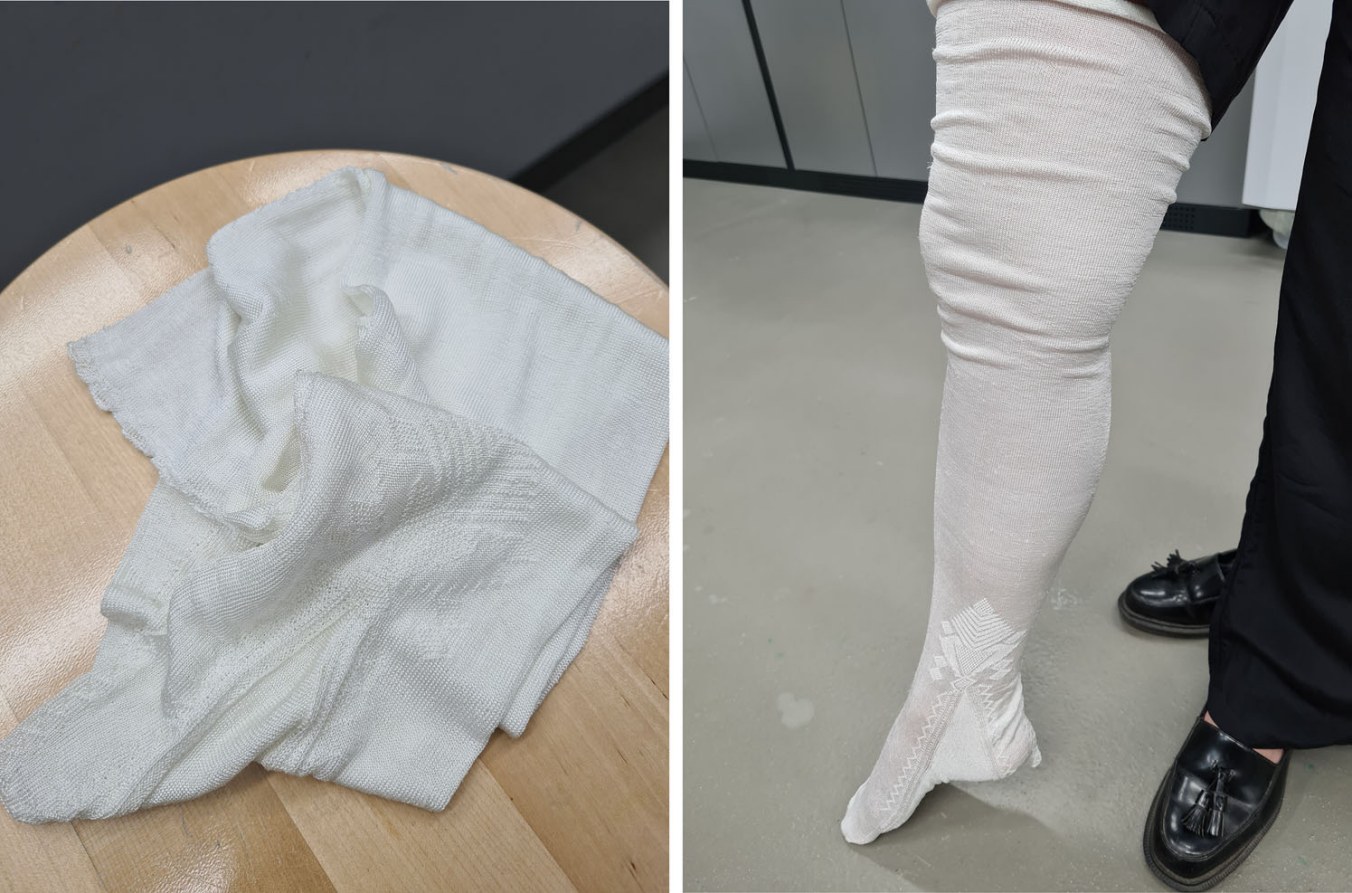
Reconstruction of the Turku silk stocking. 1364d, Bombyx mori silk, hand-reeled. © Photograph by Refashioning the Renaissance.

I started knitting a tiny little swatch with 0.7 mm needles and the thinnest wool yarn. The smallest needles I had knitted with until then were 1.75 mm and the difference seemed enormous. The needles felt really flimsy and bendy in my hands, but the result was a fabulously thin and fine piece of knitting with just over 10 loops per centimetre.^68^

After the test swatches were made, however, all volunteer knitters decided to use a set of five 1 mm needles for both silk and wool because—although these were also very thin—they were less bendy and easier to manage.

The 1 mm needles allowed most of the volunteer knitters to achieve the right gauge. The modern double-pointed steel needles used in the experiment, however, probably did not correspond fully to the early modern ‘iron rods’.^69^ The modern knitting needles used in our project might not have been as firm as the original knitting needles used in the early modern period. The tips of the needles were so sharp at both ends that many volunteer knitters reported having had to protect their fingers from cuts by using thimbles or covering the fingertips with tape. ‘After two hours of knitting’, wrote one of the knitters in her notes, ‘my right hand was tired and the fingertips were very sore’.^70^ Others found that our needles were too smooth. The stitches tended to fall off at the pointed ends of the needles because the needles were slippery.^71^ One of the participants reported that one of her needles broke in two on row 160.

The level of detail and the technical sophistication required for the knitting of such fineness made knitting fine stockings, such as the silk stocking from seventeenth-century Turku, extremely time-consuming. A statement issued in 1663 by the Vicario di Provisione, a Milanese magistrate appointed to settle guild disputes, mentioned that it took a knitter from twelve to fifteen days to knit a silk stocking by hand. In his *Dizionario delle arti de’mestieri*, written in 1768, the author Grisellini claimed that the hand-knitter needed a month to knit a pair of silk stockings.^72^

Although historians writing on early modern stockings seem to think that estimates such as those provided by the Vicario di Provisione or Grisellini of the time invested in knitting fine silk stockings are major exaggerations, the reconstruction of the seventeenth-century silk stocking from Turku Cathedral Museum suggests that these may have been not far from reality.^73^ Each of the four reconstructions of the stocking, made in silk exactly to the measure of the original stocking on 1 mm needles, took between 215–260 hours to make, meaning up to 520 hours per pair. One of the voluntary participants reported that it took her nearly three hours just to cast on the required 288 loops and to knit the first course, or round. To make the whole stocking of silk on an average speed of 2.5 hours per day, it took her in total 105 days, or 260 hours.

She wrote:

For the first 120 courses, my pace was roughly thirty minutes per course. As the stocking progressed, slowly but surely, I managed to pick up a little speed. Normally I am a fairly fast knitter, but with the bendy needles and tiny stitches it was impossible to knit without concentrating all the time. No ‘flow’ like with a normal sock, just toiling away focusing on the knitting.^74^

Another knitter reported that the speed of her knitting for the leg part was between 20–30 minutes per course in natural daylight, but the work progressed much more slowly when it was dark and she had to knit in artificial light.^75^ Some found it especially slow to knit the decorative clocking pattern, because, as one of the knitters said, ‘it was difficult to follow the pattern at the same time as I had to keep the tiny purl stitches tight’ (Fig. 16).^76^ Even considering that the early modern professional knitters were probably able to knit much faster than our knitters, all of whom were experienced and highly skilled knitters but not professionals nor used to knitting fine silk or historical patterns, it is unlikely that even a skilled early modern hand-knitter would have been able to produce multiple pairs of fine silk stockings in a month.

The reconstruction of the Turku stocking in wool showed that knitting stockings of fine wool was slightly faster than working with silk. Knitting the Turku stocking using fine wool instead of silk took approximately 135 hours to knit on 1 mm needles.^77^ Several participants of the reconstruction project reported, however, that they found knitting with fine wool, in fact, at times more laborious than with silk, both because the spun wool yarn split more easily than the long slightly twisted silk filaments, and because picking up slipped stitches was more difficult. After working with thin wool yarn, one of the volunteer participants said, ‘I often fell into trouble with a slipped stitch, a mistake to correct or just the furry yarn going into an unidentifiable mess’.^78^

Due to the time-consuming process of fine knitting in both silk and wool, it is possible that the labour was divided between several knitters. The easier, repetitive parts might have been knitted by less skilled labourers, children, elderly people, or workshop assistants, while the technically more challenging parts that contained many decreases and increases, such as the decorative pattern at the ankle, were possibly completed by the more experienced and skilled knitting master. A technical examination of the silk stocking in Turku Cathedral Museum showed that there is a change in the pattern of the false zigzag back seam at the height of the clock, suggesting that different parts of the stockings might have been knit by more than one pair of hands.

The time and skill invested in knitting made the labour costs of fine stockings relatively high. The surviving account books of Niccolo Capponi, a member of one of Florence’s oldest and wealthiest families, show that, for example, on 1 August 1571, he paid the knitter (*acucchiatore*) Antonio Bernardo 14 *lire* for making ‘a pair of stockings of local silk’ and another 13 *soldi* and 4 *denari* for processing and finishing them.^79^ The cost of the labour is equivalent to an entire two weeks’ salary of a highly skilled master artisan. Capponi’s accounts show, furthermore, that the costs for making knitted stocking of high-quality fine wool were also high. In 1571, he paid one cap-maker (*berrettaio*), Girogio, 9 *lire* and 10 *soldi* for ‘making a pair of stockings of wool *stame*’.^80^

Compared to both the simple wool socks and stockings that were produced in mass quantities by peasants, children, and labourers with minimal skill as well as the cloth stockings made by tailors, finely knit stockings were valued pieces. According to Capponi’s accounts, commissioning tailored stockings of wool cloth cost just a fraction of that of the knitter’s work. In 1571, for example, Capponi paid his tailor Napoleone less than ½ *lire* (9 *soldi* and 4 *denari*) for ‘making a pair of hose of green wool cloth’.^81^ The labour costs for tailored stockings of wool, then, were less than five per cent of the costs for making knitted fine wool stockings and only about three per cent of the costs of the labour of knitting silk stockings. This confirms that knitting fine stockings was labour intensive, and constituted a significant proportion of the value, cost, and prestige of the knitted leg garments.

## Bringing Stockings to ‘Life’

During the Refashioning the Renaissance participatory knitting reconstruction project, in total four replicas of the Turku stocking in silk were fully completed. In addition, two reconstructions in wool were made for comparative purposes. The finished silk stockings were initially rough and bulky, because they were made using degummed silk yarn in order to make the knitting easier (Fig. 17). The right shape and smooth texture were not achieved until the finishing processes had been carried out.^82^ After the glue-like sericin had been removed from the silk and the stocking had been shaped and dried on a wooden sock-block, the finished pearl white silk reconstructions were surprisingly even despite the flaws in the silk yarn, and the stockings had an incredibly soft, silky texture. The stockings were transparent and fine, weighing as little as 30.1 grams, and the clock pattern, reflecting light and standing out from the knitted fabric in relief, added to the splendour of the look (Figs 18 and 19).^83^

**Fig. 20.**
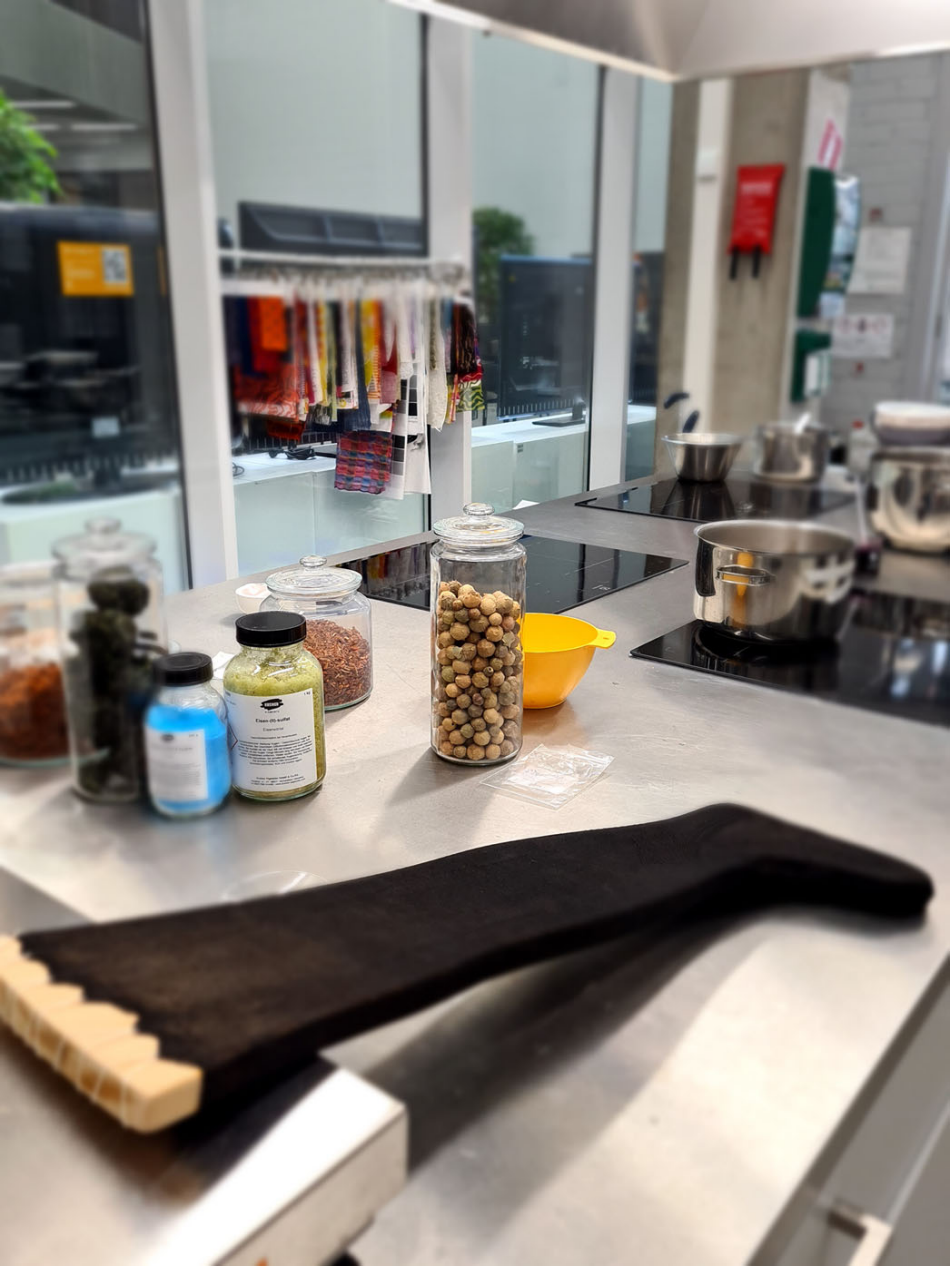
The Refashioning the Renaissance reconstruction of the Turku silk stocking 1364d. Bombyx mori silk, iron-mordanted, dyed with logwood. © Photograph by Refashioning the Renaissance.

**Fig. 21.**
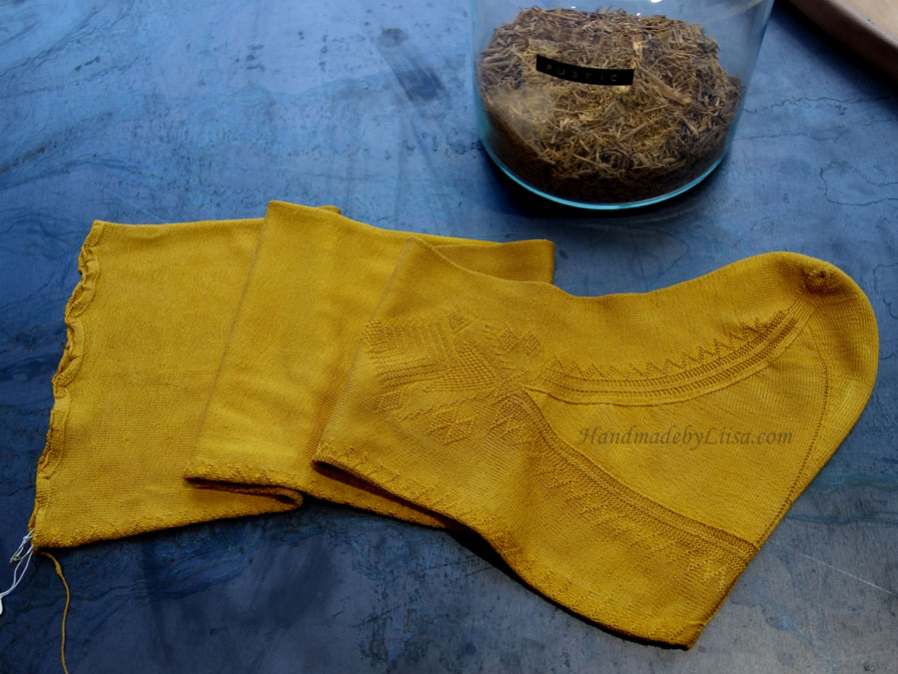
Reconstruction by Liisa Kylmänen of the seventeenth-century silk stocking at Turku Cathedral Museum, Refashioning the Renaissance Project, 2020 (inventory number CSS008), dyed with fustic on alum-mordanted silk. © Photograph by Liisa Kylmänen.

**Fig. 22.**
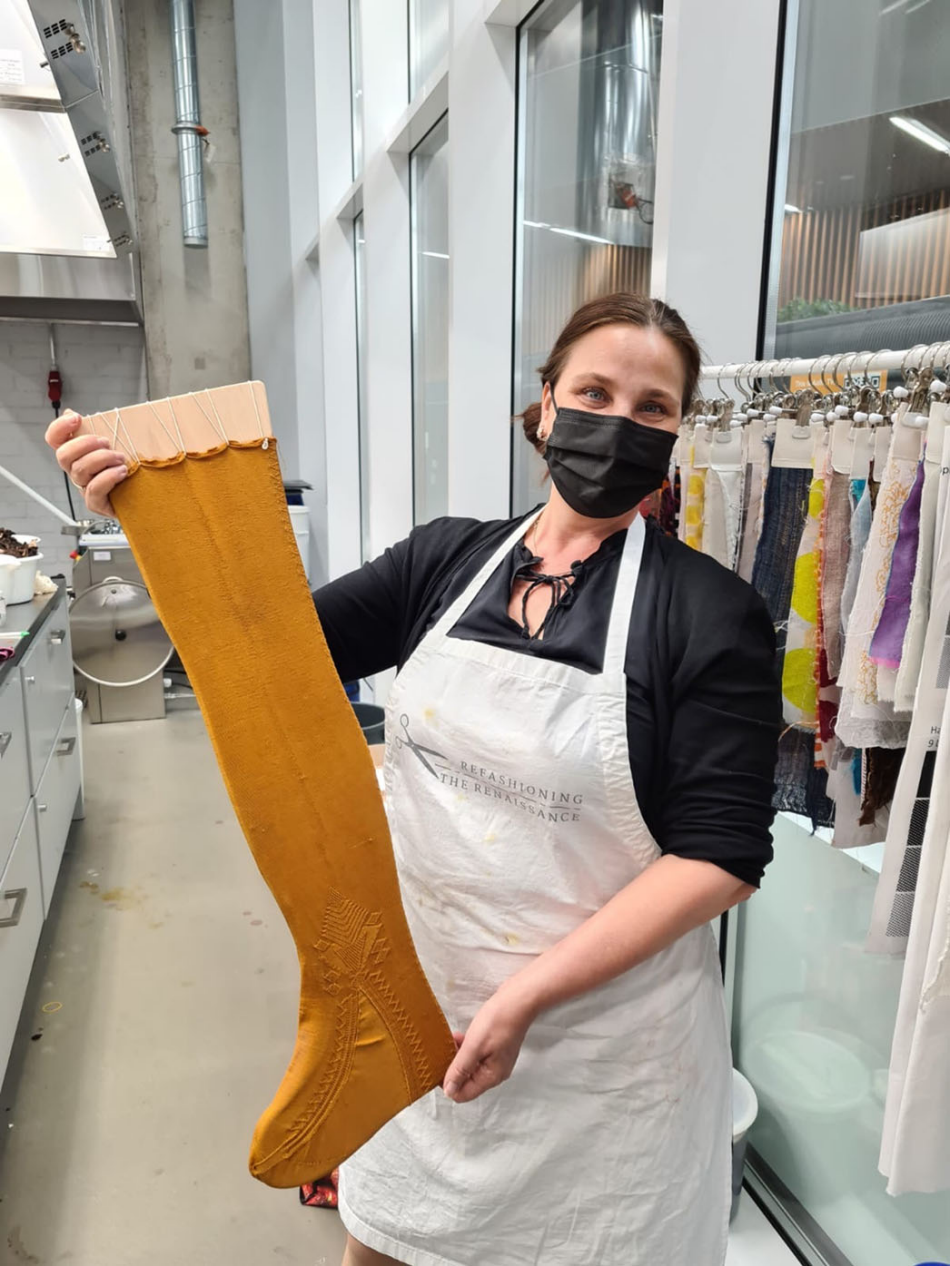
Turku silk stocking reconstruction dyed with fustic. Photograph by the Refashioning the Renaissance.

Since most stockings in this period were dyed in bright colours, typically in shades of yellow, red, green, blue, violet, white, grey, pink, and black, dyeing the stockings with appropriate colours allowed us to imagine what the invisible stockings in our fragmentary visual, archival, and material records may have looked like originally. ^.^An analysis of the organic colorants of the surviving Turku silk stocking, carried out by the Cultural Heritage Agency of the Netherlands, showed that, although the present colour of the silk stocking is a dull brown, the stocking originally had been black, dyed with tannin-rich organic materials and mordanted with an iron source.^84^ Fine black was one of the colours of high fashion in this period and garments dyed in black were an expensive commodity, due to the complex dye process of obtaining true black.^85^ The original method identified in the chemical dye analysis—possibly the ‘lasagna’ method that was made of layers of alderbark, iron swarf, iron sulphate and water in a vat and allowed to stand for at least twenty-four days before using—was too complex to carry out within the university setting.^86^ We brought the original black colour back to life by using logwood, the period’s new dye source for black, which provided a saturated black but was less resistant to light (Fig. 20).^87^ The transparent fine quality of the black silk stocking made it look similar to the very fine black sixteenth-century stocking depicted, for example, in the image of a hosier painted by Jacopo Coppi in 1580–1585, found today in the Alberto Bruschi da Grassina collection.^88^ Another silk stocking was dyed in golden yellow, a typical colour found for silk stockings in this period in our dataset and also seen in visual culture (Fig. 5), using natural dyes and standardised historical methods based on recipes from dye manuals of the period such as the *Plichto*. This recipe was based on fustic and it was dyed on alum-mordanted silk (Figs 21 and 22).^89^

Since silk was treasured not only for its look but also for its sensory qualities, an essential part of the experiment was to try on the stocking and to experience how the garment felt on the body (Fig. 19). Although we cannot, as Sarah Bendall notes, ‘accurately capture all the nuances of sensations or ideas of comfort in the same way as people five hundred years ago’, three members of our team who tried on the delicate knitted silk stocking agreed that the instant bodily sensation of wearing the finely knitted silk next to the skin was incredibly light and soft, and provided an ultimate sense of comfort.^90^ Although a reconstruction, as Jenny Tiramani has rightly pointed out, is an ‘act of interpretation’ and never a precise copy, what made our experience of wearing, touching, and looking at the reconstructed silk stocking uniquely close to the authentic is that the actual silk material was produced by hand using the same breed of silkworms and the same methods and simple local tools that were used in silk production in Italy in the early modern period.^91^ Even the mulberry leaves that were used to feed the silkworms originated from trees that have been growing on the Calabrian soil for centuries.

Authenticity in reconstruction is unattainable. Janet Arnold has summarised the challenges in the following way:

Even if fabrics hand-woven from natural fibres are used, modern threads are spun by machine and are more even than threads spun before the Industrial Revolution. The uneven quality of hand-spun threads reflects light in a way that regular machine-spun threads cannot. The tension of the spinning and the thickness of modern sewing threads made from natural fibres also vary from those made by hand, or earlier machines, so that even if the stitches are copied, the effect cannot be exactly the same.^92^

In the case of our stocking reconstruction, however, the historical accuracy associated both with the silk material itself as well as with the way it was produced meant that our visual and physical experience was less distorted by the difficulties usually associated with the pursuit of authenticity in reconstruction.

## Conclusion

The growing popularity and aesthetic appreciation connected with knitted stockings is evident across sixteenth- and seventeenth-century historical visual and written evidence. But learning about making and wearing knitted stockings by observation, hands-on experimentation, and bodily experience allowed us to gain access to the sense of visual, material, and physical splendour associated with early modern knitted stockings in a way that cannot be fully articulated nor captured in books, archival sources, or visual images alone.^93^

This experiential ‘knowledge’, combined with evidence from traditional historical sources, makes it much clearer why stockings were so highly priced and why both high-ranking elites as well as some of those lower down the social scale were prepared to spend their savings to possess at least one pair of fashionable silk stockings. The visual and material elegance of the stockings—their look, physical form, colours, elasticity, and the value of labour associated with them—also made it much more understandable why the sixteenth-century Queen Elizabeth of England, having tried knitted silk stockings for the first time in her life, declared: ‘Henceforth I will wear no more [stockings of] cloth’.^94^

